# P-wave duration is a predictor for long-term mortality in post-CABG patients

**DOI:** 10.1371/journal.pone.0199718

**Published:** 2018-07-11

**Authors:** Sheila Tatsumi Kimura-Medorima, Ana Paula Beppler Lazaro Lino, Marcel P. C. Almeida, Marcio J. O. Figueiredo, Lindemberg da Mota Silveira-Filho, Pedro Paulo Martins de Oliveira, Otavio Rizzi Coelho, José Roberto Matos Souza, Wilson Nadruz, Orlando Petrucci, Andrei C. Sposito

**Affiliations:** 1 Cardiology Division, State University of Campinas (Unicamp), Campinas, São Paulo, Brazil; 2 Surgery Department, State University of Campinas (Unicamp), Campinas, São Paulo, Brazil; Universita degli Studi di Roma La Sapienza, ITALY

## Abstract

Risk stratification in secondary prevention has emerged as an unmet clinical need in order to mitigate the Number-Needed-to-Treat and make expensive therapies both clinically relevant and cost-effective. P wave indices reflect atrial conduction, which is a sensitive marker for inflammatory, metabolic, and pressure overload myocardial cell remodeling; the three stimuli are traditional mechanisms for adverse clinical evolution. Accordingly, we sought to investigate the predictive role of P-wave indices to estimate residual risk in patients with chronic coronary artery disease (CAD). The cohort included 520 post-Coronary Artery Bypass Grafting patients with a median age of 60 years who were followed for a median period of 1025 days. The primary endpoint was long-term all-cause death. Cubic spline model demonstrated a linear association between P-wave duration and incidence rate of long-term all-cause death (p = 0.023). P-wave >110ms was a marker for an average of 425 days shorter survival as compared with P-wave under 80ms (Logrank p = 0.020). The Cox stepwise regression models retained P-wave duration as independent marker (HR:1.37; 95%CI:1.05–1.79,p = 0.023). In conclusion, the present study suggests that P-wave measurement may constitute a simple, inexpensive and accessible prognostic tool to be added in the bedside risk estimation in CAD patients.

## Introduction

In primary prevention setting, cardiovascular risk stratification is largely accepted as an approach to select individuals in whom medical attention must be intensified[1–3]. Similar to the primary spectrum, individuals at secondary prevention present a broad range of cardiovascular risk; however, worldwide guidelines classify them as a single high-risk category[4]. Recent evidences suggest the use of risk stratification as a strategy to mitigate the Number-Needed-to-Treat and make expensive therapies both clinically relevant and cost-effective[5]. Hence, risk stratification in secondary prevention has emerged as a paramount and unmet clinical need.

Residual risk stratification in individuals with coronary artery disease (CAD) has highlighted undertreated conditions such as diabetes or dyslipidemia or the presence of target-organ injuries[6]. In individuals under optimal medical treatment, various biomarkers and the use of cardiovascular imaging exams have predicted the residual risk[7–9]. Nevertheless, most of these methods are not routinely performed in clinical practice even in developed countries.

Recently, both cardiovascular and all-cause mortality were shown to be predictable by P-wave duration in a robust clinical cohort[10]. The atrial delayed-conduction reflects inflammatory and metabolic cell remodeling that antecedes noticeable atrial enlargement, whose main stimulus is chronic pressure overload[11, 12]. In animal models and autopsies, P-wave duration relates to early histological signs of fibrosis and inflammation[12, 13]. Further into the atrial overload phase, atrial structural and functional changes identified by MRI still correlate with the P-wave changes[14]. Hence, we sought to investigate the predictive role of P-wave indices in the estimation of residual risk of patients with stable CAD who underwent to coronary bypass grafting (CABG). Our findings support the use of this simple affordable tool in clinical settings.

## Methods

**Fig 1:**
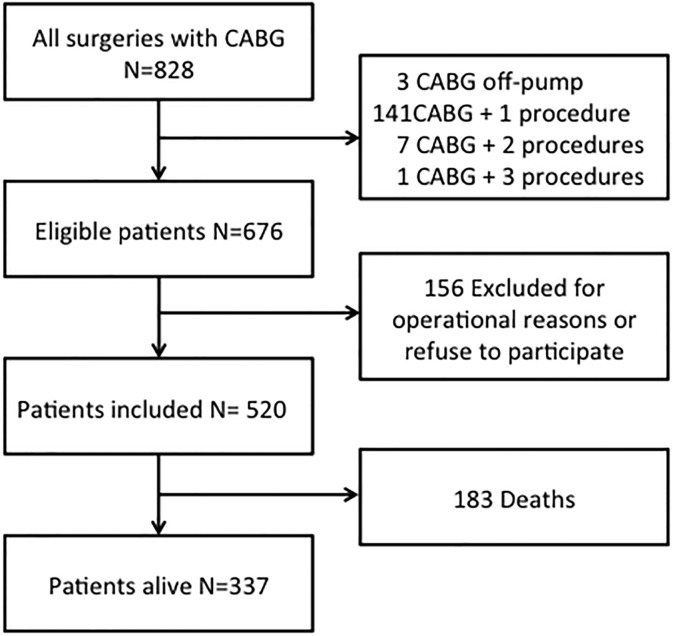
Study flow chart.

Between 2007 and 2013, we collected data from 520 consecutive patients who underwent CABG during their hospitalization at the Clinics Hospital of the State University of Campinas (HC-UNICAMP), Brazil. We included patients who underwent isolated completed CABG and excluded those who required other concomitant surgical procedure such as valve replacement and ventricular geometric reconstruction to reduce heterogeneity. These patients were selected for having complete myocardial revascularization and therefore equally asymptomatic for myocardial ischemic disease. In addition, these exclusion criteria were created with the purpose of homogenizing the population studied and concentrating the outcomes of coronary origin. The study flow chart is presented in Fig 1. The cohort recruitment for this observational study was determined to allow a minimum of 645-days follow-up period, so the vital status was accessed by telephone. All enrolled patients gave permission to participate signing the informed consent and the Institutional Ethics Committee approved this study (CAAE Nr.0828.0.146.000–10); identity and personal data are confidential.

**Fig 2:**
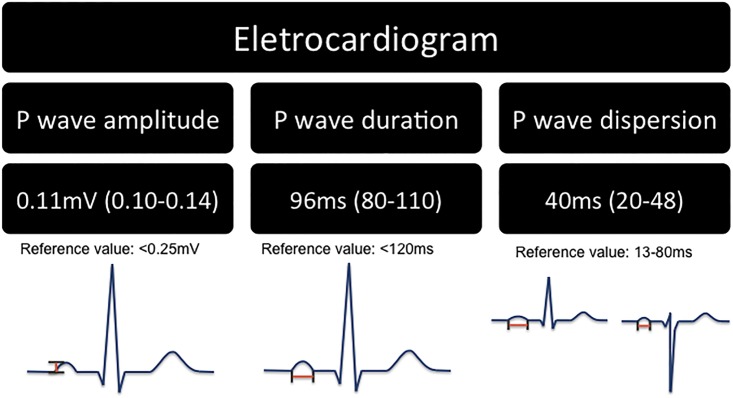
P-wave measurements and reference values.

All clinical data were measured, except for ethnic group that was self-reported. The twelve leads ECG were performed within one week from the CABG and were manually analyzed about rhythm and P-wave indices (duration, amplitude and dispersion). P-wave dispersion was calculated by subtracting the maximum and minimum P-wave durations in any of the twelve ECG leads, while exams measured P-wave duration and amplitude in lead II. We decided to use the lead II due to the fact that this lead often presents the largest P-wave duration[15]. The paper speed used was 25 mm/sec. Two experienced cardiologists (STKM and APBLL) who were blinded to the patients clinical status used manual caliper for measuring P-wave duration and had an intra-observer correlation coefficient of 0.549, p = 0.002 and 0.759, p<0.0001; and inter-observer correlation coefficient of 0.735, p<0.0001. The hand held caliper measurement were confirmed in a subset of patients by the use of electronic digital paquimeter and we found an agreement of 95.7% (standard deviation 0.133) and 98.5% (SD 0.097), respectively. The above-mentioned P-wave measurements and their reference values are shown in Fig 2

Laboratory analyses included automated blood cell count, urea (kinetic U.V. test), creatinine (Jaffe method with compensation, kinetic colorimetric test) and electrolytes (ion-selective electrode). Glomerular filtration rate (GFR) was estimated with MDRD formula[16]. Experienced physicians analyzed coronarography; a significant lesion was considered if more than 70% stenosis. Echocardiography analysis followed guidelines[17]. Chambers diameters measured in parasternal long axis M-mode. Left ventricular mass was measured according to the cube formula and left ventricular hypertrophy was considered if the mass was over 95g/m2 in women or 115g/m2 in men[17]. Ejection fraction reported as continuous variable was estimated by Teicholz method or by Simpson method when left ventricular wall motion abnormalities were observed.

The primary endpoint was long-term mortality; otherwise follow-up was censored at the last outpatient visit registered by the hospital system. Descriptive statistics of continuous and categorical data are expressed as the median and 25th and 75th percentile or frequencies and percentages, respectively. Univariate comparisons before matching and correlations among P-wave indices used Mann-Whitney test and linear regression, as appropriate. Furthermore, we applied Cox regression analysis to investigate the predictive relevance of ECG parameters to the time to all-cause mortality. Log rank tested unadjusted mortality differences according to P-wave duration quartiles in Survival Kaplan-Meier plots. Covariates included in multivariable models were pre-selected from a stepwise model, and included sex, age, and diabetes.

Spline-based model curve assessed the predictive capacity of P-wave duration for long-term all-cause death, adjusting for age, sex, diabetes, chronic obstructive pulmonary disease, stroke, two or more CABGs, creatinine, prior acute coronary syndrome episode, ejection fraction, PASP, myocardial infarction in the previous 90 days. Finally, the sample size provided post-hoc power of 100% to the primary endpoint (reference population according to Bradshaw et al.[18]). Significance level was a two-sided p-value<0.05. Analyses were performed using the Statistical Package for Social Sciences, version 21.0, software (IBM Corp, Armonk, NY) and STATA version 14.0 (StataCorp, College Station, TX).

## Results

Table 1 summarizes the cohort’s baseline characteristics. The median follow-up period was 1025 days (34 months), ranging from 645 to 2816 days. During the follow up period there were 183 (35%) deaths. Although all censured deaths were reported as secondary to cardiovascular disease, due to limited adjudication information, we only considered total mortality.

**Table 1:** Demographic characteristics.

Characteristic	No / median	% / IQR
Age (IQR)—yr	61	(54–68)
Male sex—no. (%)	375	(72.1)
Caucasian	430	(82.7)
African descendent	84	(16.1)
Asian descendent	6	(1.1)
Body-mass index (IQR)	27.9	(25.3–30.7)
Resting heart rate (IQR)—bpm	67	(61–76)
Medical history—no. (%)		
Obesity	159	(30.6)
Diabetes	219	(42.1)
Hypertension	450	(86.5)
Previous myocardial infarction	378	(72.7)
Previous stroke	31	(6.0)
Previous surgical revascularization	19	(3.7)
Previous percutaneous coronary intervention	59	(11.3)
Previous atrial fibrillation	16	(3.1)
Any other arrhythmia	18	(3.5)
Chronic Obstructive Pulmonary Disease	34	(6.5)
Active or former smoker—no. (%)	326	(62.7)
Symptoms		
No angina	148	(28.4)
Angina CCS Class 4	104	(20.0)
Medication—no. (%)		
ACE inhibitor or ARB	417	(80.2)
Betablocker	436	(83.8)
Calcium-channel blocker	146	(28.1)
Diuretic	171	(32.9)
Statin	498	(95.8)
Echocardiogram		
Left atrium (IQR)—mm	40	(38–43)
Left ventricle (IQR)—mm		
Diastolic diameter (IQR)—mm	52	(50–55)
Ejection fraction (IQR)—%	60	(48–67)
PASP (IQR)—mmHg	30	(30–30)
Left ventricle hypertrophy—no. (%)	292	(56.2)
Mitral regurgitation—no. (%)	295	(56.7)
Mitral stenosis—no. (%)	5	(1.0)
Aortic regurgitation—no. (%)	99	(19.0)
Aortic stenosis—no. (%)	14	(2.7)
Diastolic dysfunction—no. (%)	325	(62.5)
Preoperative coronarography—no. (%)		
Anterior Descendent Coronary Artery stenosis	519	(99.8)
Circumflex Coronary Artery stenosis	445	(85.6)
Right Coronary Artery stenosis	408	(78.5)
3-Vessel disease	359	(69.0)
Laboratory (IQR)		
Creatinine clearance (MDRD)—m/min/1.73m^2^	84.9	(68.8–105)
Sodium—mmol/L	141	(139–142)
Potassium—mmol/L	4.3	(4.0–4.6)
Hemoglobin—g/dL	13.9	(12.8–14.8)
Hematocrit—%	41.5	(37.8–44.1)
Leucocytes—mm^3^	7495	(6355–9012)
Electrocardiogram		
P wave amplitude (IQR)—mV	0.11	(0.10–0.14)
P wave duration (IQR)—ms	96	(80–110)
P wave dispersion (IQR)—ms	40	(20–47)
P wave duration over 110ms—no. (%)	138	(26.5)
Preoperative risk assessment		
Euroscore I (0-13points)		
Low risk (0–2 points)	210	(40.4)
Medium risk (3–5 points)	144	(27.7)
High risk (6 or more points)	166	(31.9)

**Fig 3:**
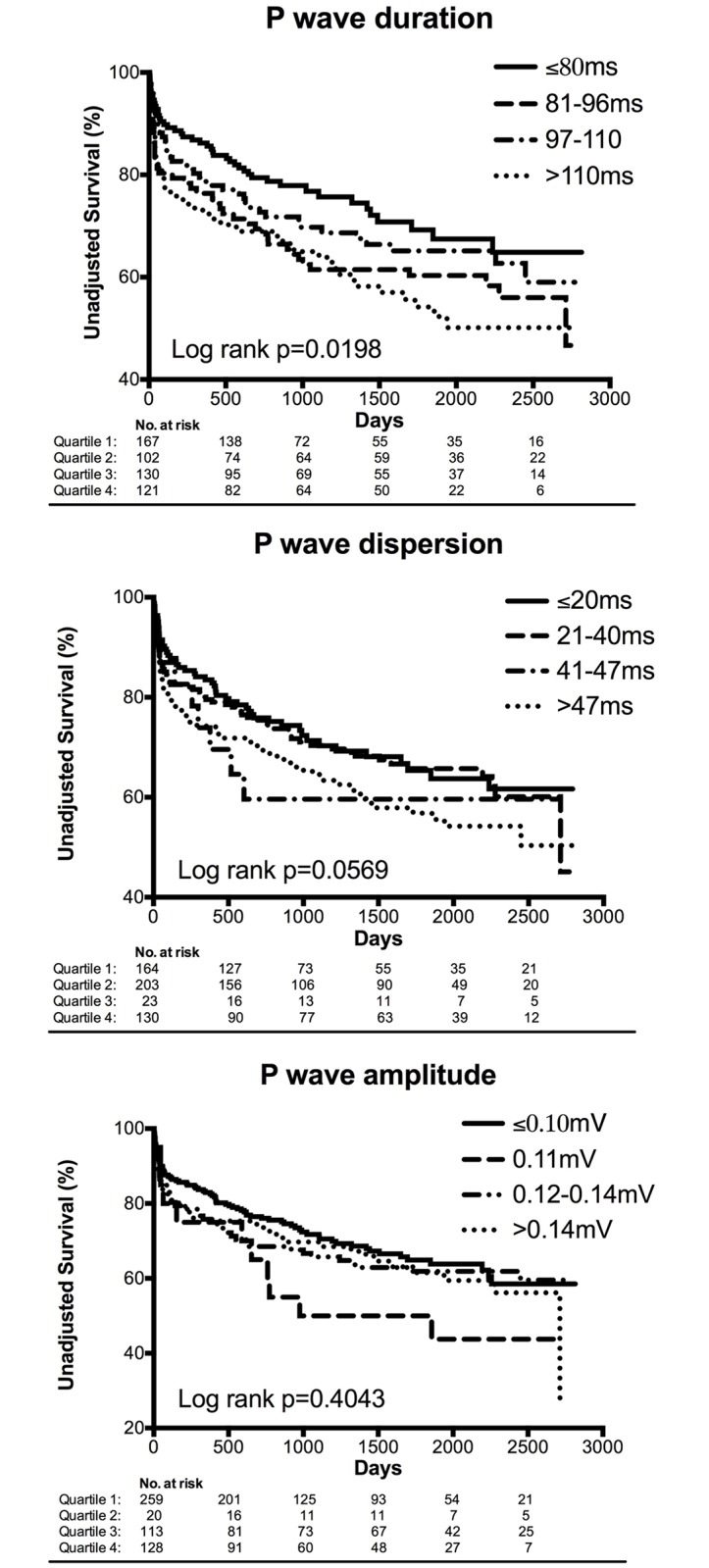
Kaplan-Meier curves for all-cause mortality, according to the P-wave indices (quartiles).

There was a linear relation between P-wave duration and P-wave dispersion (Constant 2.033, beta 0.373, p<0.001) (Figure A in S1 File). In the linear regression between P-wave indices and primary endpoint, only P-wave duration was related to mortality (β+,R-square = 0.011,p = 0.014). P-wave duration over 110ms determined an average minus 425 days of life when compared to P-wave ≤80ms (2096 vs. 1671 days; Log rank p = 0.0198). No difference was found when comparing survival curves according to P-wave amplitude or P-wave dispersion (Fig 3).

Linear regressions with P-wave parameters were performed to assess clinical correlates and pre-select variables for multivariable analysis (Table A in S1 File). Thereafter, we performed a multiple linear regression analyses using stepwise method and including variables associated to each P-wave indices (model entry P = 0.05, removal P = 0.10). Multivariable linear regression model for clinical correlates for each P-wave indices is detailed in Table 2.

**Table 2:** Multivariable linear regression for clinical correlates of each P wave indices^1^.

Characteristic	β(+/-)	SE	p-value
**P wave duration (R square = 0.080)**			
Urea	+	0.001	<0.001
Diuretic (Thiazides or Loop)	+	0.049	0.004
ARB or ECA inhibitor use	+	0.056	0.036
Angina pectoris	-	0.050	0.036
**P wave amplitude (R square = 0.090)**			
Urea	+	0.001	<0.001
Diastolic dysfunction	+	0.036	0.001
Creatinin Clearance (MDRD)	+	0	<0.001
Female	+	0.039	0.005
Caucasian	-	0.046	0.020
**P wave dispersion (R square = 0.042)**			
Previous arrythmia	-	0.115	0.005
EuroSCORE low risk	-	0.043	0.012
Sodium	+	0.006	0.018
Diastolic dysfunction	-	0.057	0.029

^1^ Linear regression using stepwise forward method including significant clincal correlates and also age, sex and diabetes.

Beta estimaes slope line.

R square estimates the model contribution to predict each P-wave indice.

**Fig 4:**
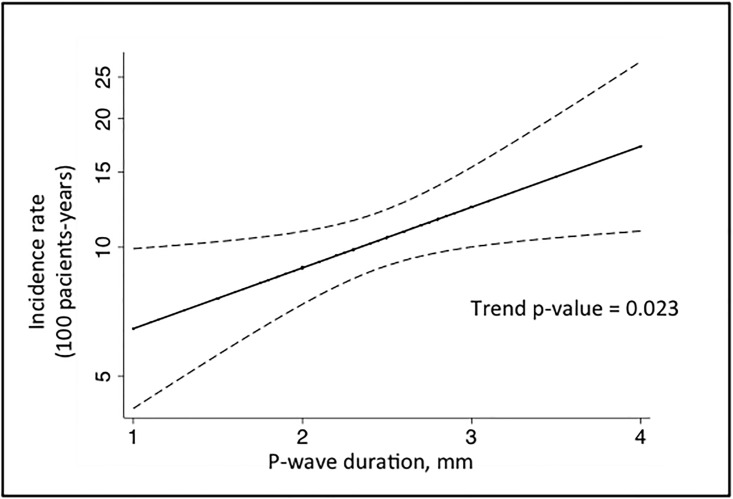
Spline model predictiveness curve for P-wave duration and the risk prediction for all-cause death. The analysis was adjusted for age, sex, diabetes, chronic obstructive pulmonary disease, stroke, two or more previous CABG, creatinine, previous acute coronary syndrome, ejection fraction, pulmonary artery systolic pressure.

Thereafter the Cox proportional hazards regression analyses were performed to identify independent predictors of long-term mortality, including ECG parameters (Table 3). The categorical variable “P-wave ≥110ms” was an independent predictor of long-term all-cause mortality, adjusted for age, sex and diabetes (HR = 1.40, 95%CI = 1.02–1.91, p = 0.036). The Cox proportional hazards regression multivariable analyses incorporating stepwise regression models including all covariables described above and all three P-wave indices (model entry p = 0.05, removal p = 0.10) retained the P-wave duration, two or more CABGs, prior acute coronary syndrome episode, pulmonary arterial systolic pressure (PASP) >60mmHg and diabetes in the final model (model p-value <0.0001, retained variables in Table 4). In a Spline curve model we observed a linear association between P-wave duration and the incidence rate of long-term all-cause death for 100-patients-years in a fully adjusted model with trend p-value = 0.023 (Fig 4). We obtained similar results when adjusting only for age, sex and diabetes (trend p-value = 0.029). Also, history of paroxysmal atrial fibrillation occurred in 18 (3.5%) before surgery but it had no interaction in the association between P-wave duration and survival.

**Table 3:** Independent predictors of death by univariate Cox regression analysis ^1^.

Characteristic	Hazard ratio	p-value	95% CI
**Baseline characteristics**			
Diabetes	1.365	**0.036**	1.021–1.824
Angina (CCS 1–4)	0.890	**0.022**	0.805–0.984
Previous atrial fibrillation	2.369	**0.008**	1.252–4.486
Diuretic use	1.361	**0.039**	1.016–1.823
Medication was discontinued >24h preoperatively			
ACE inhibitor	0.719	**0.049**	0.517–0.999
PASP—mmHg	1.025	**0.016**	1.005–1.046
Left ventricle hypertrophy	1.501	**0.009**	1.106–2.037
Preoperative laboratory			
Urea	1.012	**0.002**	1.004–1.020
Creatinin	1.147	**0.027**	1.015–1.296
Hemoglobin—g/dL	0.903	**0.020**	0.829–0.984
Hematocrit—%	0.967	**0.027**	0.939–0.996
Preoperative electrocardiogram			
P wave duration—mm	1.374	**0.022**	1.047–1.803
**Perioperative variables**			
Index surgical procedure			
Arterial grafts (0–3)	0.627	**0.023**	0.419–0.937
Total of grafts (1–4)	0.775	**0.027**	0.618–0.971
Days in Intensive Unit Care	1.018	**0.020**	1.003–1.034
**Outcomes**			
Postoperative atrial fibrillation	1.850	**0.004**	1.220–2.803
Recurrent atrial fibrillation	2.611	**<0.001**	1.536–4.439
Postoperative myocardial infarction	2.471	**0.030**	1.094–5.584

^1^ The test was performed with all valid variables.

The table shows those with p-value<0.05.

**Table 4:** Retained variables in Cox proportional hazards regression multivariable analyses^1^.

Characteristic	Hazard ratio	p-value	95% CI
P-wave duration (mm)	1.430	**0.010**	1.091–1.876
PASP over 60mmHg	5.967	**<0.0001**	2.416–14.738
Previous CABG	2.161	**0.026**	1.095–4.262
Diabetes	1.395	**0.025**	1.043–1.866
Angina CCS 4	0.632	**0.033**	0.415–0.963

^1^ Stepwise regression models including all variables from EuroSCORE I and all three P-wave indices (model entry p = 0.05, removal p = 0.10)

## Discussion

The study was designed to investigate the clinical value of using regular ECG for estimating the residual risk in individuals with stable CAD. Our main finding points an increase of 37% in the long-term relative risk of all-cause death for each mm of increase in P-wave duration.

P-wave duration might reflect the electrical remodeling of the atria and is a predictor of death, atrial fibrillation or heart failure hospitalization in a large spectrum of patients[19],[20] including those post-CABG [21]. This simple parameter is a potential marker of atrial overload preceding clinical event and is histologically correlated to the extent of fibrosis and fatty infiltration in atrial tissues[13]. Interestingly, the Bachmann’s bundle and terminal crest were the most affected areas, suggesting that these areas play a major role of inter- and intra atrial conduction on prolongation of P-wave duration[13]. In animal model, prolonged P-wave duration was related to abnormal inter-atrial conduction, independent of the left atrium size, mediated by dysregulation of connexin proteins expression (CX 40 and CX 43) and fibrosis[12]. In patients with acute coronary syndromes, it has been found an increased number of inflammatory cells infiltrate the atria, coming from the adipose tissue, suggesting that the left ventricular infarction induces atrial inflammation [22]. By inference, it is possible that the prolonged P-wave represents an early sign of chronic or acute inflammatory stimuli on atrial tissue.

P-wave duration of 154ms has been show to convey a three-fold all-cause adjusted mortality risk in a long-term prospective cohort in the general population[10]. In our study P-wave duration over 110ms was associated to all-cause adjusted long-term mortality, suggesting that this parameter should be considered in patients with CAD. In contrast, in the only prior study with post-CABG patients, P-wave duration was not related to mortality, however PR interval was a significant predictor of death after adjustment for confounders[23]. Since the three studies had prolonged clinical follow-up and adequate statistical power, the divergence of results should be due to the differences in severity of the residual risk in patients enrolled, observed for instance by the incidence of diabetes in our population (42%, compared to 27% in Lauer et al. [23]). In fact, P-wave was a risk marker in both studies with individuals at less severe cardiovascular risk. In the study with individuals at greater overall risk, P-wave was not a predictor but rather electrocardiographic signals compatible with more advanced cardiac structural alterations such as left ventricular overload[23].

Diastolic dysfunction is a marker of common pathophysiologic process related to long-term pressure overload and cardiac remodeling[24], thus P-wave duration could reflect insults from clinical or subclinical diseases and act as a noninvasive barometer of clinical status[11]. Diastolic dysfunction was significantly associated to P-wave amplitude and P-wave dispersion in our study, but did not reach significance for P-wave duration, possibly due to qualitative assessment of diastolic function instead of quantitative measurements, such as E/e’ index for example.

In spite of the fact that the P-wave indices are easily obtained the main limitation is related to measurement techniques. It was already demonstrated that hand-held calipers measurements have less accuracy compared with digital measurements[25]. However, the present study reflects the real world where most of the ECG is manually examined. Besides the possible error of measurement our data reached statistical relevance, reflected previous findings and clinical correlates, demonstrating its feasibility as a prognostic tool. We only assessed vital status and we were not able to verify differences between cardiovascular and non-cardiovascular death due to sample size and adjudication limitations. Since atrial fibrillation is often an asymptomatic arrhythmia, we weren’t able estimate the association between P-wave duration and the incidence of this arrhythmia post-discharge. P-wave axis was recently demonstrated as another potential marker for high-risk post-CABG patients[26]. This finding was not available when the present study was designed and we are able to verify the additive value of these two P-wave markers. Finally, we did not perform echocardiographic evaluation of patients enrolled. Thus, future studies are required to better characterize the nature of this association.

In secondary prevention setting the balance between the residual risk and the use of expensive new therapeutic options is a daily concern for clinicians worldwide. So far, very few studies have dedicated to identify variables that may potentially be useful for such risk discrimination, particularly those subclinical, non-invasive and non-expensive. In the present study, we found that P-wave duration may represent one of these variables and having all the above-mentioned features, we believe it must be considered in prospective multivariate modeling for generating risk algorithms in CAD patients. Meanwhile, in bedside clinical practice, finding a P-wave ≥110ms must be taken as a warning sign.

## Supporting information

S1 File Figure A. Linear regression between P-wave duration and dispersion. Table A. Clinical correlates of P-wave indices.
(DOCX)

## Abbreviations

AF: Atrial Fibrillation
CABG: Coronary Artery Bypass Grafting
CAD: Coronary Artery Disease
CCD: Congestive Cardiac Disease
COPD: Chronic Obstructive Pulmonary Disease
ECG: Electrocardiogram
EuroSCORE: European system for cardiac operative risk evaluation
GFR: Glomerular filtration rate
IDI: Integrated Diagnostic Improvement
IQR: Interquartile range
MDRD: Modification of Diet in Renal Disease Study equation
MRI: Magnetic Resonance Imaging
NRI: Net Reclassification Improvement
PASP: Pulmonary Artery Systolic Pressure
PCI: Percutaneous Coronary Intervention
