# Supplementary material for: P-wave duration is a predictor for long-term mortality in post-CABG patients
Source: PLoS One. 2018 Jul 11;13(7):e0199718. doi: 10.1371/journal.pone.0199718 (PMC6040706; doi:10.1371/journal.pone.0199718)
Supplement: S1 File — Figure A. Linear regression between P-wave duration and dispersion. Table A. Clinical correlates of P-wave indices. (DOCX) [file pone.0199718.s001.docx]

# Supplementary material

**Figure A.** Linear regression between P-wave duration and dispersion.


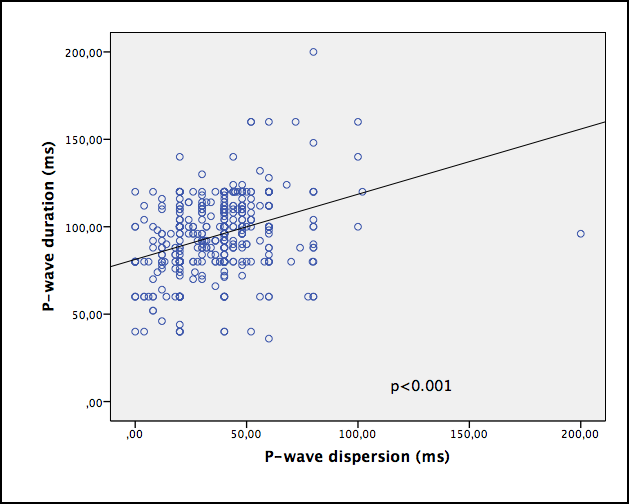


**Table A.** Clinical correlates of P-wave índices^1^.

|  | **P-wave duration** | | | **P-wave amplitude** | | | **P-wave dispersion** | | |
| --- | --- | --- | --- | --- | --- | --- | --- | --- | --- |
| **Characteristic** | **β (+/-)** | **R square** | **p-value** | **β (+/-)** | **R square** | **p-value** | **β (+/-)** | **R square** | **p-value** |
| Age | + | 0.003 | 0.208 | - | 0.004 | 0.159 | + | 0.001 | 0.395 |
| Female sex | - | 0.003 | 0.230 | + | **0.011** | **0.018** | + | 0.002 | 0.292 |
| Caucasian | - | 0.001 | 0.490 | **-** | **0.016** | **0.003** | - | 0 | 0.695 |
| African descendent | + | 0.001 | 0.489 | + | **0.015** | **0.005** | + | - | 0.917 |
| Asian descendent | + | 0 | 0.951 | + | 0.001 | 0.556 | + | 0.002 | 0.302 |
| Body-mass index | + | 0 | 0.702 | - | 0.001 | 0.402 | + | 0.002 | 0.338 |
| Heart rate (bpm) | - | 0.002 | 0.280 | + | 0 | 0.967 | + | 0 | 0.856 |
| Diabetes | + | 0.001 | 0.443 | + | 0.001 | 0.513 | + | 0.003 | 0.206 |
| Hypertension | + | 0.003 | 0.234 | - | 0 | 0.964 | - | 0 | 0.848 |
| Hypothyroidism | + | 0 | 0.973 | + | 0.001 | 0.394 | + | 0 | 0.652 |
| Previous myocardial infarction | + | 0.006 | 0.079 | + | 0.004 | 0.136 | + | 0.001 | 0.523 |
| Previous surgical revascularization | - | 0.001 | 0.441 | - | 0.001 | 0.441 | - | 0.001 | 0.543 |
| Previous percutaneous coronary intervention | - | 0 | 0.753 | - | 0.001 | 0.583 | - | 0 | 0.912 |
| Angina (CCS) 4 | **-** | 0 | 0.771 | - | 0 | 0.736 | + | 0.002 | 0.375 |
| Angina pectoris | **-** | **0.014** | **0.007** | + | 0 | 0.951 | - | 0.003 | 0.194 |
| Arrhythmia |  |  |  |  |  |  |  |  |  |
| Previous atrial fibrillation | - | 0 | 0.933 | - | 0 | 0.745 | + | 0 | 0.975 |
| Any other arrhythmia | - | 0.004 | 0.130 | - | 0.001 | 0.404 | **-** | **0.013** | **0.010** |
| Pulmonary obstructive chronic disease | + | 0.002 | 0.360 | - | 0.001 | 0.387 | + | 0 | 0.838 |
| Active or former smoker | - | 0.001 | 0.580 | + | 0.003 | 0.208 | + | 0.001 | 0.539 |
| Medication |  |  |  |  |  |  |  |  |  |
| ACE inhibitor or ARB | **+** | **0.009** | **0.028** | + | 0 | 0.823 | + | 0 | 0.711 |
| Betablocker | - | 0 | 0.766 | - | 0 | 0.666 | **-** | **0.009** | **0.031** |
| Calcium-channel blocker | + | 0.002 | 0.351 | - | 0 | 0.959 | - | 0.001 | 0.405 |
| Diuretic (Thiazides or Loop) | **+** | **0.025** | **<0.001** | + | 0.004 | 0.148 | + | 0 | 0.786 |
| Statin | + | 0.001 | 0.606 | + | 0.001 | 0.202 | - | 0.002 | 0.376 |
| Echocardiogram |  |  |  |  |  |  |  |  |  |
| Left atrium - mm | + | 0.005 | 0.116 | **-** | 0 | 0.636 | + | 0.001 | 0.609 |
| Left atrium - over 40mm | **+** | **0.010** | **0.026** | - | 0 | 0.923 | + | 0 | 0.621 |
| LV Diastolic diameter - mm | **+** | **0.012** | **0.014** | - | 0.001 | 0.543 | + | 0.001 | 0.439 |
| LV Ejection fraction - % | **-** | **0.011** | **0.018** | - | 0.002 | 0.277 | - | 0.004 | 0.141 |
| PASP - mmHg | - | 0.001 | 0.567 | + | 0 | 0.859 | + | 0.002 | 0.316 |
| Left ventricle hypertrophy | - | 0.001 | 0.582 | + | 0.004 | 0.130 | + | 0.001 | 0.499 |
| Diastolic dysfunction | + | 0.005 | 0.114 | **+** | **0.025** | **<0.001** | **+** | **0.009** | **0.033** |
| Preoperative coronarography |  |  |  |  |  |  |  |  |  |
| Anterior Descendent Coronary Artery stenosis | - | 0.003 | 0.231 | - | 0.002 | 0.364 | - | 0.003 | 0.218 |
| Circumflex Coronary Artery stenosis | **+** | **0.009** | **0.027** | + | 0 | 0.846 | - | 0 | 0.788 |
| Right Coronary Artery stenosis | + | 0 | 0.675 | - | 0.004 | 0.133 | - | 0 | 0.950 |
| 3-Vessel disease | + | 0.004 | 0.164 | - | 0.002 | 0.266 | - | 0 | 0.903 |
| Laboratory |  |  |  |  |  |  |  |  |  |
| Urea | **+** | **0.046** | **<.0001** | **+** | **0.021** | **0.001** | + | 0 | 0.651 |
| Creatinine | + | 0.001 | 0.552 | + | 0.001 | 0.495 | - | 0 | 0.939 |
| Creatinine Clearance (MDRD (mL/min/1,73m2) | + | 0.001 | 0.416 | **+** | **0.010** | **0.022** | + | 0 | 0.790 |
| Sodium - mmol/L | + | 0.006 | 0.081 | + | 0.001 | 0.446 | **+** | **0.008** | **0.042** |
| Potassium - mmol/L | - | 0 | 0.770 | + | 0 | 0.794 | - | 0 | 0.975 |
| Hemoglobin - g/dL | - | 0.007 | 0.060 | - | 0.005 | 0.106 | + | 0.002 | 0.315 |
| Hematocrit - % | **-** | **0.008** | **0.036** | - | 0.004 | 0.147 | + | 0.003 | 0.246 |
| Preoperative risk assessment |  |  |  |  |  |  |  |  |  |
| EuroSCORE I (range 0-13 points) | + | 0.002 | 0.360 | + | 0.001 | 0.446 | + | 0.006 | 0.082 |
| Low risk (0-2 points) | - | 0 | 0.896 | - | 0.002 | 0.301 | **-** | **0.008** | **0.047** |
| Medium risk (3-5 points) | - | 0.002 | 0.301 | + | 0 | 0.814 | + | 0.005 | 0.106 |
| High risk (6 or more points) | + | 0.002 | 0.264 | + | 0.001 | 0.392 | + | 0.001 | 0.595 |
|  | | | | | | | | | |

^1^Unadjusted linear regression analysis. Beta estimates slope line. R square estimates the variable contribution to predict each P-wave indices.
